# Beneficial Effects of a Low-Glycemic Diet on Serum Metabolites and Gut Microbiota in Obese Women With *Prevotella* and *Bacteriodes* Enterotypes: A Randomized Clinical Trial

**DOI:** 10.3389/fnut.2022.861880

**Published:** 2022-05-02

**Authors:** Haeng Jeon Hur, Xuangao Wu, Hye Jeong Yang, Min Jung Kim, Kyun-Hee Lee, Moonju Hong, Sunmin Park, Myung-Sunny Kim

**Affiliations:** ^1^Food Functionality Research Division, Korea Food Research Institute, Wanju, South Korea; ^2^Obesity/Diabetes Research Center, Department of Food and Nutrition, Hoseo University, Asan, South Korea; ^3^Department of Food Biotechnology, Korea University of Science and Technology, Wanju, South Korea

**Keywords:** low-glycemic diet, fecal bacteria, insulin resistance, metabolites, enterotypes, low-cholesterol diet, 3-hydroxybutyric acid

## Abstract

Generalized healthy eating patterns may not benefit everyone due to different genetics and enterotypes. We aimed to compare the effects of a low-glycemic diet representing the Korean traditional balanced diet (Low-GID) and westernized diet as a control diet (CD) on anthropometry, serum metabolites, and fecal bacteria in a randomized clinical trial according to enterotypes. We recruited 52 obese women aged 30–50 years, and they consumed Low-GID and CD meals for 1 month, with a 1-month washout period, in a crossover randomized clinical trial. The Low-GID was mainly composed of whole grains with fish, vegetables, seaweeds, and perilla oil, whereas CD contained refined rice, bread, noodles, meats, and processed foods. Serum lipid profiles, metabolomics, serum short-chain fatty acids, and fecal bacteria were analyzed. The important variables influenced by Low-GID and CD were determined by SHAP value in the XGBoost algorithm according to *Bacteroides* (ET-B) and *Prevotella* (ET-P). Low-GID and CD interventions did not change the enterotypes, but they modified serum metabolites and some fecal bacterial species differently according to enterotypes. The 10-fold cross-validation of the XGBoost classifier in the ET-P and ET-B clusters was 0.91 ± 0.04 and 0.8 ± 0.07, respectively. In the ET-P cluster, serum L-homocysteine, glutamate, leucine concentrations, and muscle mass were higher in the CD group than in the Low-GID group, whereas serum 3-hydroxybutyric acid concentration was significantly higher in the Low-GID group than in the CD group (*p* < 0.05). In fecal bacteria, *Gemmiger formicilis, Collinsella aerofaciens*, and *Escherichia coli* were higher in the CD group than in the Low-GID group. In the ET-B cohort, serum tryptophan and total cholesterol concentrations were higher in the CD group than in the Low-GID group, whereas serum glutathione and 3-hydroxybutyric acid concentrations were significantly higher in the Low-GID group than in the CD group (*p* < 0.05). However, *Bifidobacterium longum* was higher in CD than Low-GID in the ET-B cluster, but serum butyric acid levels were higher in the Low-GID than in the CD group. In conclusion, Low-GID can be recommended in obese women with both ET-P and ET-B enterotypes, although its efficacy was more effective in ET-P.

**Clinical Trial Registration:**

[https://cris.nih.go.kr/cris/search/detailSearch.do/17398], identifier [KCT0005340].

## Introduction

Various microorganisms exist in all human body parts, including the intestines, mouth, and skin. Most of these microorganisms are located in the large intestines with more than 4,000–10,000 different microorganisms, representing 3.5 million genes, much greater than human genes (1). The gut microbiota bidirectionally interact with humans as a host to influence human health (2). Age, gender, diet, nutrient intake, and lifestyles in the host modulate the gut microbiota community (2). Gut microbiota affect the host’s aging, immunity, food digestion, and drug functions and produce various metabolites that affect the host’s metabolism (1). Gut dysbiosis has been reported to be related to obesity, type 2 diabetes, dyslipidemia, Alzheimer’s disease, autism, depression, and other diseases (3, 4). Dietary modification can maintain gut eubiosis to prevent metabolic diseases.

South Korea has developed its own characteristic diet for a long period (5). Like the Mediterranean diet, the health benefits of the Korean traditional balanced diet have also been paid attention to in promoting healthy diets in various countries. The Korean diet is characterized by a high proportion of whole grains, cooked vegetables, beans, seaweeds, fish, fermented beans, and vegetables (5). The health benefits of the Korean traditional balanced diet are potentially related to its cooking methods, diverse foods, and cooking oil, including sesame and perilla oil (5). According to the previous studies, the Korean traditional balanced diet has low glycemic, antioxidant, and anti-inflammatory properties (6, 7). With socioeconomic growth in South Korea, this traditional diet pattern has undergone a rapid transformation toward westernized diets (8), which are mainly composed of animal meat, processed foods, refined rice, flour, and sugar and are generally considered unhealthy, leading to metabolic diseases, such as obesity, diabetes, and atherosclerosis (9, 10).

People with different ethnicities have major enterotypes because they share similar long-term lifestyles, mainly diet patterns (11). Since changes in individual gut microbiota are challenging to study, human enterotypes need to be divided into three categories: *Prevotella*, *Bacteroides*, and *Ruminococcus* (12). According to the previous studies, the *Prevotella* enterotype is related to a high intake of carbohydrates, especially sugar, whereas the *Bacteroides* enterotype is involved in high meat intake (11). There are a few studies on the connection between the *Ruminococcus* enterotype and diet, where some have pointed out that *Ruminococcus* and *Bacteroides* enterotypes are not the discrete clusters but continuums (13). The Mediterranean diet is one of the world’s recognized healthy diets with antioxidant and anti-inflammatory properties (14). However, Huttenhower et al. showed that the Mediterranean diet has a better effect on reducing the risk of cardiometabolic diseases in individuals without *Prevotella copri* in the gut, supporting the need for a personalized diet based on gut microbiota (15).

Obesity is growing in Asians, and it is related to modifying the dietary patterns to consume more refined rice, processed foods, meats, and less vegetables with economic growth. The dietary changes of diet may alter the gut microbiota related to increasing obesity incidence. The traditional Korean balanced diet may have a protective effect on obesity, but there is no randomized clinical study about its impact on weight loss by modulating gut microbiota according to the enterotypes. Therefore, we aimed to determine the weight loss effect of the traditional Korean diet, representing a low-glycemic and low-cholesterol diet, and westernized Korean diet on gut microbiota and metabolomics in obese women aged 30–50 years according to enterotypes, such as *Bacteroides* and *Prevotella*.

## Materials and Methods

### Participants and Experimental Design

The Institutional Review Board approved the study protocol at CHA University (1044308-201801-HR-033-04), and the study was conducted under the Declaration of Helsinki. All participants provided written informed consent, and it was registered at the Korean Clinical Trial Registry (CRIS; trial number KCT0005340). We voluntarily recruited 54 obese women aged 30–50 with a body mass index (BMI) of 23 ≤ body mass index ≤ 30. They did not take diabetes, hyperlipidemia, hypertension, or hyperlipidemia medications, and they did not have a food allergy. The study started on 4 November 2019 and ended on 28 May 2020, and this clinical study was conducted using a crossover design study.

The sample size was calculated based on a previous human study (16): a difference in the inflammatory index ≥1.3 was clinically significant at a level of α = 0.05, power of 0.80, and standard deviation of 0.80. The sample size was 48 for each group in the crossover design trial, and participants were added considering a 10% dropout rate. The initial sample size was 54 participants in each group, and one participant from each group dropped out due to not signing for the informed content form.

In the first trial, 52 participants were randomly divided into two groups (each group *n* = 26) with random numbers generated by a computer, and they were randomly and blindly allocated into westernized diet (control diet, CD) and Korean traditional balanced diet representing low-glycemic and low-cholesterol diet (Low-GID) groups with random numbers. They consumed the assigned diet for 4 weeks and then had a 4-week washout period. In the second trial, the diet was switched to the other diet, and the participants consumed their newly assigned diet (either CD or Low-GID) for 4 weeks, followed by a 4-week washout period. Low-GID and CD were prepared for 1,900 kcal/day, an estimated energy requirement for 30–50 women. According to the Low-GID and CD meal plans, meals with beverages and snacks were delivered to each participant’s home. Participants were encouraged to maintain their lifestyle, physical activity level, and diet throughout the study period. However, alcohol consumption was restricted.

### Experimental Diet

Each Low-GID and CD meal was cooked under the supervision of a dietician. The composition of the Low-GID and CD is presented in Table 1 and complies with the Koreans’ dietary reference intake. The calorie intake for each participant was approximately 1,900 kcal per day, the estimated energy requirement for one Korean woman aged 30–49 years. Both diet patterns were based on Korean meal types, including staple food, soup, kimchi, and three side dishes in each meal (5). The Low-GID included two servings of whole grains as a staple food, 1–2 servings of fish or poultry, two servings of cooked vegetables, one serving of kimchi, and one serving of fruits. They were cooked using traditional Korean cooking methods, such as boiling, steaming, blanching, and pickling. CD consisted of two servings of refined rice, bread, or noodles as staple foods, one to two servings of meats, two servings of uncooked vegetables, one serving of kimchi, and one serving of fruits. The Low-GID had tea, fruit tea, and soymilk while CD had coffee, juice, and milk. They were cooked fried, stir-fried, sautéed, or baked, and uncooked vegetables were served with salad dressing.

**TABLE 1:** Food and nutrient intake during the intervention.

Food group	Low-GID (*n* = 52)	CD (*n* = 52)	*p*-value^1^
Total grains (g)	240 ± 2.6^1^	195 ± 8.73	<0.0001
Whole grains (g)	196.8 ± 2.2	1.7 ± 0.72	<0.0001
Vegetables (g)	380 ± 2.9	261 ± 12.3	<0.0001
Fruits (g)	90.4 ± 0.9	86.9 ± 5.05	NS
Kimchi (g)	171 ± 4.2	109 ± 13.0	<0.0001
Fermented soybeans (g)	22.2 ± 0.2	12 ± 0.72	<0.0001
Soybeans and tofu (g)	76.8 ± 0.6	16.8 ± 1.44	<0.0001
Fishes and seafood (g)	57.8 ± 0.7	26.1 ± 1.44	<0.0001
Meats (g)	12.9 ± 0.1	30.9 ± 1.44	<0.0001
Seaweeds (g)	7.4 ± 0.1	4.8 ± 0.72	<0.0001
Nuts (g)	7.4 ± 0.1	2.3 ± 0.72	<0.0001
Perilla oil (g)	5.7 ± 0.1	0.3 ± 0.72	<0.0001
Glycemic index of meal	50.3 ± 3.55	68.1 ± 2.89	<0.0001
Energy (kcal)	1834 ± 106	1859 ± 60.5^1^	0.1385
Carbohydrates (En %)	67.2 ± 0.50	62.4 ± 0.40	<0.0001
Dietary fiber (g)	36.7 ± 2.22	21.3 ± 0.83	<0.0001
Protein (En %)	13.0 ± 0.17	14.0 ± 0.10	<0.0001
Fat (En %)	19.5 ± 0.36	24.1 ± 0.33	<0.0001
Saturated fatty acids (En %)	2.45 ± 0	6.3 ± 0.48	<0.0001
MUFA (En %)	2.94 ± 0	5.3 ± 0.48	<0.0001
PUFA (En %)	4.91 ± 0.49	5.3 ± 0.48	NS
Cholesterol (mg)	146 ± 9.32	272 ± 7.84	<0.0001

*Values are presented as means ± standard deviations.*

*^1^Statistical analysis with a two-sample t-test.*

*Low-GID, a low-glycemic diet representing Korean traditional balanced diet; CD, westernized diet; En %, energy percent; MUFAs, monounsaturated fatty acids; PUFAs, polyunsaturated fatty acids.*

The glycemic index (GI) of foods in the Low-GID and CD was extracted from the International GI Table in 2021 (17). GI values of foods from Asian countries were used to calculate the GI of the meals. GI of the daily meal was calculated by the following equation: GI for daily intake = $\sum_{k=1}^{n}{\left(\mathrm{each}\mathrm{food}\mathrm{of}\mathrm{GI}\times \mathrm{carbohydrate}\mathrm{intake}\mathrm{from}\mathrm{the}\mathrm{food}\right)}_{\text{k}}/\mathrm{total}\mathrm{carbohydrate}\mathrm{intake}\mathrm{for}\mathrm{one}\mathrm{day})$ (18). K indicated the number of foods consumed for a day. Means and standard deviations were calculated for 28-day meals. A computer-assisted nutritional analysis program (CAN-pro-3.0; Korean Nutrition Society, Seoul, South Korea) was used to calculate the energy and nutritional intake of the 24-h intervention meal. The Low-GID was characterized by 65–68% carbohydrates (energy), 15% protein, and 17–20% fat. The CD was composed of 60–63% carbohydrates, 15% protein, and 24–26% fat. Table 1 lists the two diets and their calorie intake.

### Primary Outcome Measurements

The measurements were taken in the Metabolic Research Unit of the Korea Food Research Institute. Primary outcome measurements included anthropometry and metabolites, including lipid profiles in the circulation. BMI was calculated by dividing body weight by height squared (kg/m^2^). Muscle mass and fat mass were measured using InBody 4.0 (Cheonan, South Korea). Blood pressure was assessed using an automatic blood pressure monitor on the left arm. Total cholesterol, high-density lipoprotein (HDL), low-density lipoprotein (LDL), triglycerides, and plasma glucose levels were measured using a Hitachi 7600 Autoanalyzer (Hitachi Ltd., Tokyo, Japan). Serum high-sensitive C-reactive protein (CRP) and insulin concentrations were analyzed using an ELISA kit (Thermo Fisher Scientific, Waltham, MA, United States).

For metabolomic analysis, pretreated serum was mixed with cold acetonitrile and shaken for 30 min at 4°C. The supernatants were separated after centrifugation at 6,000 × *g* for 10 min. The supernatant was collected, and 20% caffeine methanol solution was added. The metabolites of the mixture were measured using ultraperformance liquid chromatography and quadrupole time-of-flight (UPLC-Q-TOF) method using an Acquity UPLC System (Waters, Milford, MA, United States). The stationary phase was Acquity UPLC BEH C18 (2.1 mm × 100 mm, 1.7 μm) chromatographic column, and the mobile phase was an aqueous solution and acetonitrile solution containing 0.1% formic acid. The separation program temperature was 40°C, and the flow rate was 0.35 ml/min (19).

Serum short-chain fatty acid (SCFA) measurement was based on adding a solvent mixture of n-butanol, tetrahydrofuran, and acetonitrile (Duksan, Ansan, South Korea) to the serum. The serum was completely vortexed and mixed for 1 min, separated at the top of the supernatant after centrifugation at 7,500 × *g*, 15 min, and 4°C, and the separated upper layer was filtered with a 0.45-μm microporous filter. After filtration, it was injected into a PerkinElmer Clarus 680 GAS Chromatograph (Waltham, MA, United States). The stationary phase was an Elite-FFAP 30 m × 0.25 mm × 0.25 μm capillary column, and the mobile phase was high-purity helium. The column temperature program was established by increasing from 100 to 180°C at 10°C/min and then by elevating from 180 to 220°C at 20°C/min. The hydrogen, air, and helium flow rates were 45 ml/min, 450 ml/min, and 20 ml/min, respectively (20).

### Secondary Outcomes

The fecal bacteria community was measured by next-generation sequencing (NGS). We used the QIAamp Power Fecal DNA Kit (12830-50; QIAGEN, Germany) to extract total DNA from feces. Using the KAPA HiFi HotStart Ready Mix PCR Kit (KK2602; Kapa Biosystems, United States), amplification was performed under the following conditions of PCR: 94°C for 3 min; 35 cycles at 94°C for 15 s, 55°C for 45 s, and 72°C for 1 min; and then extended at 72°C for 8 min. The 16S rRNA universal amplification primers used were rRNA genes B341F (5′-CCTACGGGNGGCWGCAG-3′) and B805R (5′-GACTACHVGGGTATCTAATCC-3′) (20). Electrophoresis was conducted on an agarose gel to confirm amplification. The amplified 16S rRNA was purified using AMPure beads (Beckman Coulter, Brea, CA, United States), and its head and tail were labeled using the Nextera XT Index Kit (Illumina, San Diego, CA, United States). The sample was subjected to the following PCR conditions: 95°C for 3 min; 8 cycles at 95°C for 30 s, 55°C for 30 s, and 72°C for 30 s; 72°C for 5 min; and then kept at 4°C. The labeled DNA was purified and sent to Macrogen Inc. (Seoul, South Korea) for sequencing.

### Fecal Bacterial Community Analysis

After running MiSeq, 204 pairs of paired-end sequencing files were obtained, with 5,000,352 paired-end original sequences. Using the Mothur program (version 1.43.0) (21), 2,500,176 sequences were obtained after merging the double-ended sequences with the make.contigs command, the sequences were aligned with the SILVA v 1381 database, and non-target sequences, such as mitochondria, archaea, fungi, and unknown sequences, were removed. The remaining sequences were preclustered, and chimeras were eliminated using the chimera.vsearch command. The sequences were then clustered, with 97% similarity. The generated FASTA files were annotated according to the Greengenes reference taxonomy (22). Finally, 40,289 representative sequences were obtained for the subsequent analyses.

The correlation between serum concentration of SCFA (acetic acid, propionic acid, and butyric acid) and normalized fecal bacteria at the genus level was assessed using the Hmice package in the R program according to the Low-GID and CD.

### Safety Outcome Measurements

Clinical conditions, including adverse reactions, were evaluated before and after interventions. Serum alanine aminotransferase (ALT), aspartate aminotransferase (AST), white blood cells (WBCs), and platelet concentrations were measured.

### XGBoost Classifier Training and Shapley Additive Explanations Interpreter

To study the difference between the Low-GID and the CD effects according to enterotypes *Bacteroides* (ET-B) and *Prevotella* (ET-P), we used the Python-based XGBoost (version 1.3.3) package to train the characteristic features of the Low-GID and CD as classification devices in the ET-P and ET-B clusters (23). The characteristic features included the relative abundance of fecal bacteria at the species level, anthropometric variables, serum biochemical, and metabolic variables. A random grid search was used to find the best hyperparameter settings, and the number of search times was 1,000 in the XGBoost algorithm (24). We first trained the XGBoost algorithm with all variables to find the top 10 most important variables and then used these ten variables to retrain the XGBoost algorithm. Because the sample size was small, we used all of them to train the classifiers (Low-GID and CD) and then used 10-fold cross-validation to evaluate the accuracy of the selected model. The 10-fold cross-validation was calculated using the cross_val_score function in the sklearn package. The function splits the original training data into ten subsets and alternately used nine of them as training data and one as test data for iterating ten times. Finally, ten sets of data were generated to obtain the mean and variance, which were used as the final accuracy result of the model. Then, 0.9 of 10-fold cross-validation indicated that the accuracy of the selected model was 90%.

Shapley Additive exPlanations is a method used to explain the output of the XGBoost model (25). We used the SHAP (0.39.0) package to calculate the SHAP value of each variable relative to the classifier (diet types) and observed the importance of the variable and its impact on the classification.

The separation of Low-GID and CD groups in the included variable of the XGBoost model was determined by principal component analysis (PCA) in total participants, ET-B, and ET-B using FactoMineR and Factoextra packages in the R program.

### Statistical Analysis

Mothur was used to perform downstream analysis of fecal bacteria; for β-diversity measurement, clearcut command in Mothur was used to construct a phylogenetic tree, unifrac.unweighted command was applied to calculate the unweighted UniFrac distance matrix, and then principal coordinate analysis (PCoA) was used for visualization. Analysis of molecular variance (AMOVA) command was conducted to compare a significant difference among β-diversity groups. The α-diversity metric was calculated with a summary.single command in the Mothur, and the Chao1 and Shannon indices were obtained. The enterotype analysis of each participant was acquired from the website^1^ (12). Linear mixed model analysis was performed to analyze the differences between the Low-GID and CD in a crossover design study using the LME function of the NLE package of R-studio, and the ggplot2 package was used for visualization. The data are expressed as the mean ± standard deviation (SD), and statistical significance was set at *p* < 0.05.

## Results

### General Characteristics of the Participants According to Enterotypes

Age was significantly different by enterotypes of the participants but not by dietary types (Table 2). Anthropometric measurements, such as body weight, BMI, waist circumferences, lean body mass, and fat mass, were not significantly different from the dietary types and enterotypes of the participants (Table 2). Metabolic parameters that include blood pressure, white blood cell counts, serum triglyceride, total cholesterol, HDL, LDL, glucose, insulin, and CRP concentrations did not differ by dietary types and enterotypes of the participants (Table 2). Also, the Homeostatic model assessment for insulin resistance (HOMA-IR) and HOMA for β-cell function (HOMA-B) were not different.

**TABLE 2:** Baseline characteristics in a low-glycemic diet (Low-GID) representing Korean traditional balanced diet and westernized diet as a control diet (CD) according to enterotypes.

	*Prevotella* enterotype		*Bacteroides* enterotype	
Variable	Low-GID (*n* = 27)	Control-diet (*n* = 26)	Low-GID (*n* = 25)	Control-diet (*n* = 26)
Age (years)	40.3 ± 1.04^a^	40.4 ± 1.01^a^	37 ± 1.08^b^	37 ± 1.11^b^**
Weight (kg)	65.3 ± 1.16	64.7 ± 0.97	65.6 ± 1.35	65.9 ± 1.5
Body mass index (kg/m^2^)	25.4 ± 0.36	25.2 ± 0.32	25.5 ± 0.32	25.6 ± 0.38
Waist circumferences (cm)	79.6 ± 1.26	78.9 ± 1.07	81.2 ± 1.16	81.7 ± 1.09
Lean body mass (kg)	21.8 ± 0.43	21.7 ± 0.38	22 ± 0.5	22.4 ± 0.53
Fat mass (%)	38 ± 0.72	38.2 ± 0.71	38.4 ± 0.75	38.2 ± 0.85
Systolic blood pressure (mmHg)	118 ± 2.34	112 ± 2.46	116 ± 2	116 ± 1.98
Diastolic blood pressure (mmHg)	72.5 ± 1.27	73.2 ± 1.82	75 ± 1.24	74.9 ± 1.29
White blood cell (×10^3^/μl)	5.85 ± 0.31	5.69 ± 0.23	5.86 ± 0.24	5.62 ± 0.28
Platelet (×10^3^/μl)	24.6 ± 0.9	23.9 ± 0.77	25.6 ± 1.05	26.8 ± 0.97
Serum triglyceride (mg/dL)	90.3 ± 9	93.9 ± 9.47	111 ± 12.3	99 ± 11.7
Serum total cholesterol (mg/dL)	188 ± 6.25	187 ± 6.39	183 ± 5.81	178 ± 6.15
Serum HDL-C (mg/dL)	61.2 ± 2.88	61.7 ± 3.2	59.7 ± 2.64	60.5 ± 3.28
Serum LDL-C (mg/dL)	109 ± 5.82	106 ± 5.41	101 ± 5.95	98.3 ± 5.77
Fasting plasma glucose (mg/dL)	97 ± 1.21	97.2 ± 1.65	98.7 ± 1.62	97.9 ± 1.52
Fasting plasma Insulin (mU/L)	10.2 ± 0.72	9.37 ± 0.75	10.6 ± 0.72	10.3 ± 0.69
HOMA-IR	2.46 ± 0.18	2.26 ± 0.19	2.58 ± 0.21	2.54 ± 0.19
HOMA-B	110 ± 7.48	100 ± 6.99	112 ± 7.43	108 ± 7.33
Serum CRP (mg/dL)	1.13 ± 0.12	1.25 ± 0.23	2.82 ± 1.89	2.16 ± 0.62

*Values are presented as means ± standard deviation.*

*HDL-C, high-density lipoprotein cholesterol; LDL-C, low-density lipoprotein cholesterol; HOMA-IR, homeostatic model assessment for insulin resistance; HOMA-B, HOMA for β-cell function; CRP, high-sensitive C-reactive protein.*

**Significantly different by enterotype using two-way ANOVA with an interaction term at p < 0.01.

*^a,b^Means in the same row with different superscript letters were significantly different by Tukey’s test at p < 0.05.*

### Primary Outcomes According to Enterotypes

Body mass index was lower in the Low-GID group than in the CD group in both enterotypes. However, muscle mass was lower in the Low-GID group than in the CD group. In the ET-P cluster, muscle mass was significantly higher in the Low-GID group than in the CD group (*p* < 0.05) (Table 3).

**TABLE 3:** Anthropometric and serum biochemical index of the participants according to *Prevotella* and *Bacteroides* enterotypes after the intervention of a low-glycemic diet (Low-GID) representing Korean traditional balanced diet and westernized diet as control diet (CD).

	*Prevotella* enterotype			*Bacteroides* enterotype		
	Low-GID (*n* = 27)	CD (*n* = 26)	*p*-value^1^	Low-GID (*n* = 25)	CD (*n* = 26)	*p-*value^2^
Body mass index	24.7 ± 0.33	25.1 ± 0.33	<0.0001	25.0 ± 0.37	25.3 ± 0.37	0.04
Waist circumferences (cm)	77.6 ± 1.04	79.1 ± 1.05	0.16	78.6 ± 1.02	79.8 ± 1.01	0.16
Muscle mass (%)	21.5 ± 0.38	21.9 ± 0.38	<0.0001	21.9 ± 0.48	22.0 ± 0.48	0.58
Body fat (%)	37.6 ± 0.66	37.3 ± 0.66	0.17	37.2 ± 0.88	37.6 ± 0.87	0.49
Serum total cholesterol (mg/dL)	160 ± 5.25	173 ± 5.29	0.001	153.8 ± 5.80	169.4 ± 5.76	0.007
Serum triglyceride (mg/dL)	74.9 ± 7.76	90.93 ± 7.84	0.018	71.8 ± 7.44	87.0 ± 7.35	0.04
Serum HDL (mg/dL)	52.0 ± 2.60	56.9 ± 2.62	0.005	51.2 ± 1.77	55.1 ± 1.75	0.005
Serum LDL (mg/dL)	92.8 ± 4.48	97.8 ± 4.53	0.16	88.3 ± 5.42	96.9 ± 5.39	0.02
Atherogenic index^1^	2.20 ± 0.141	2.19 ± 0.142	0.87	2.07 ± 0.15	2.18 ± 0.15	0.29
Serum CRP (mg/dL)	1.11 ± 0.03	1.18 ± 0.16	0.36	1.71 ± 0.40	1.20 ± 0.40	0.37
HOMA-IR	1.58 ± 0.14	1.85 ± 0.12	0.08	1.78 ± 0.21	1.79 ± 0.20	0.97
HOMA-B	82.2 ± 6.88	95.7 ± 7.00	0.11	81.6 ± 6.85	88.7 ± 6.73	0.47
QUICKI	0.36 ± 0.005	0.35 ± 0.005	0.12	0.36 ± 0.005	0.36 ± 0.004	0.40

*Values are presented as means standard deviation.*

*HDL, high-density lipoprotein cholesterol; LDL, low-density lipoprotein cholesterol; CRP, C-reactive protein; HOMA-IR, homeostatic model assessment for insulin resistance; HOMA-B, HOMA for β-cell function; QUICKI, quantitative insulin-sensitivity check index; atherogenic index: the ratio of HDL and LDL.*

*^1,2^Statistical difference between K-diet and CD in Prevotella enterotype^1^ and Bacteroides enterotype^2^ using a linear mixed model for crossover design.*

In both ET-P and ET-B clusters, serum total cholesterol and triglyceride concentrations were lower in the Low-GID group than in the CD group. However, serum LDL cholesterol concentrations in the Low-GID group were lower than those in the CD group in ET-B, but not in the ET-P group (*p* < 0.05) (Table 3). The atherogenic index calculated by serum HDL and LDL cholesterols did not differ between the Low-GID and CD in both enterotypes. Furthermore, there was no significant difference in serum CRP concentrations, HOMA-IR, HOMA-B, and quantitative insulin-sensitivity check index (QUICKI) between the Low-GID and CD in both enterotypes (Table 3). However, HOMA-IR tended to be lower in the Low-GID group than in the CD group in the *Prevotella* enterotype (*p* = 0.08).

In the ET-P cluster, serum concentrations of leucine, branched-chain amino acids (BCAAs), γ-glutamylisoleucine, tryptophan, tyrosine, glycine, L-homocysteine, and uric acid were lower in the Low-GID group than in the CD group (Table 4). In contrast, serum isoleucine and 3-hydroxybutyric acid concentrations in the Low-GID group were higher than those in the CD group (*p* < 0.05) (Table 4). In the ET-B cluster, serum BCAA, tryptophan, and uridine concentrations were significantly lower in the Low-GID group than in the CD group, whereas serum glutathione and 3-hydroxybutyric acid concentrations in the Low-GID group were higher than those in the CD group (*p* < 0.05). Serum SCFA concentrations did not differ between the Low-GID and CD in both enterotypes, but serum butyric acid concentrations were higher in the Low-GID than in the CD only in the ET-B cluster (Table 4).

**TABLE 4:** Serum metabolites of the participants according to *Prevotella* and *Bacteroides* enterotypes after a low-glycemic diet (Low-GID) representing Korean traditional balanced diet and westernized diet as a control diet (CD).

		*Prevotella* enterotype			*Bacteroides* enterotype		
		Low-GID (*n* = 27)	CD (*n* = 26)	*P-*value^1^	Low-GID (*n* = 25)	CD (*n* = 26)	*P-*value^2^
Amino acid	Valine	42.2 ± 5.99	53.1 ± 6.11	0.08	37.2 ± 5.29	47.5 ± 5.21	0.08
	Leucine	279 ± 12.4	316 ± 12.6	0.01	262 ± 11.5	284 ± 11.3	0.10
	Isoleucine	14.9 ± 1.67	13.7 ± 0.47	0.02	13.0 ± 0.42	12.4 ± 0.42	0.16
	BCAA*^a^*	337 ± 13.5	381 ± 13.8	0.01	313 ± 12.4	343 ± 12.3	0.03
	γ-glutamylisoleucine	45 ± 6.41	65 ± 6.58	0.34	43.5 ± 6.99	52.0 ± 6.87	0.36
	Glutamate	9.00 ± 2.65	16.3 ± 2.71	0.07	7.82 ± 3.03	14.8 ± 2.98	0.07
	Glutamine	227 ± 7.32	236 ± 7.44	0.23	226 ± 8.99	222 ± 8.87	0.66
	Arginine	415 ± 57.8	531 ± 59.0	0.16	344 ± 49.0	391 ± 48.0	0.46
	Tryptophan	7,526 ± 253	8,607 ± 257	0.001	7,359 ± 215	7,849 ± 212	0.02
	Tyrosine	823 ± 29.9	971 ± 30.4	0.001	892 ± 34.8	911 ± 34.2	0.64
	Glycine	19.4 ± 0.78	23.1 ± 0.80	0.0003	19.0 ± 1.06	20.2 ± 0.91	0.30
	L-homocysteine	21.6 ± 16.8	73.3 ± 17.0	0.003	31.5 ± 16.8	43.2 ± 16.6	0.41
	Creatine	2187 ± 112	2331 ± 114	0.31	2071 ± 206	2381 ± 202	0.30
	Glutathione	43.6 ± 5.36	29.5 ± 5.47	0.08	39.8 ± 5.25	23.9 ± 5.16	0.04
Carnitines	Carnitine	9,572 ± 841	11,694 ± 857	0.09	9,637 ± 848	10,685 ± 832	0.35
	Short acylcarnitine*^b^*	1,796 ± 198	1,979 ± 202	0.52	1,684 ± 180	1,729 ± 176	0.83
	Long acylcarnitine*^c^*	38,716 ± 3390	37,184 ± 3428	0.75	36,333 ± 3824	37,388 ± 3757	0.23
Nucleosides	Uric acid	11,601 ± 424	12,986 ± 432	0.03	11,690 ± 518	12,071 ± 512	0.40
	Uridine	110 ± 5.45	119 ± 5.57	0.25	107 ± 4.24	121 ± 4.19	0.003
Organic acid	2-ketobutyric acid	199 ± 16.9	238 ± 16.9	0.12	202 ± 16.4	221 ± 16.2	0.39
	Pyruvic acid	218 ± 7.66	230 ± 7.75	0.08	214 ± 8.28	227 ± 8.17	0.15
	3-hydroxybutyric acid	15.0 ± 2.78	5.51 ± 2.82	0.002	17.4 ± 2.66	8.20 ± 2.62	0.02
	Isocitric acid	5,876 ± 213	5,983 ± 216	0.54	5,720 ± 226	5,979 ± 223	0.24
SCFA	Acetic acid	137 ± 12.9	145 ± 12.9	0.49	141 ± 8.9	135 ± 16.3	0.69
	Propionic acid	14.3 ± 0.8	14.7 ± 1.07	0.74	13.5 ± 0.63	13.4 ± 1.18	0.94
	Butyric acid	11.1 ± 0.52	10.8 ± 0.55	0.57	12.4 ± 0.64	10.6 ± 0.68	0.06

*^a^BCAA is obtained by adding valine, leucine and isoleucine.*

*^b^Short is obtained by adding propionyl carnitine, isobutyryl carnitine, isovaleryl carnitine, valeryl carnitine, and hexanoyl carnitine.*

*^c^Long is obtained by adding palmitoyl carnitine and oleoyl carnitine.*

*BCAA, branched-chain amino acids; short: acylcarnitines of short-chain fatty acid; long: acylcarnitines with long-chain fatty acid.*

*^1,2^Statistical difference between K-diet and CD in Prevotella enterotype^1^ and Bacteroides enterotype^2^ using a linear mixed model for crossover design.*

### Secondary Outcomes: Changes of Enterotypes

**FIGURE 1:**
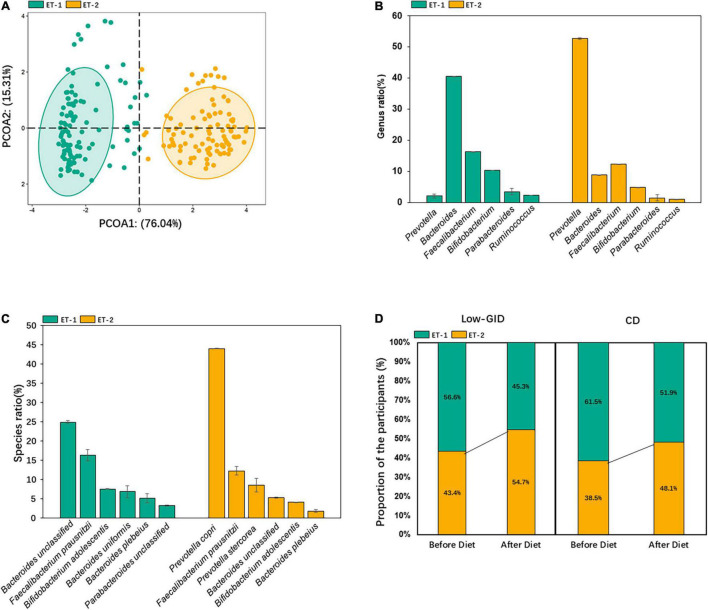
Classification of enterotypes in the participants. **(A)** Classification map based on the principal coordinates of enterotype classification at the genus level. **(B)** The relative abundance of dominant bacteria at the genus level of each enterotype. **(C)** The relative abundance of dominant bacteria at the species level of each enterotype. **(D)** Changes of enterotype after the intervention of host diet.

We used the relative abundance at the genus level to distinguish enterotypes. The fecal bacteria were separated into two enterotypes, ET-1 and ET-2 (Figure 1A). The dominant bacteria in the ET-1 group were *Bacteroides* enterotype (ET-B). *Bacteroides* mainly included *Bacteroides uniformis* and unclassified *Bacteroides*, which included various *Bacteroides* species but were not identified. The most abundant dominant bacteria at the genus level in the ET-2 group were *Prevotella* (ET-P). In ET-P, the most dominant *Prevotella* was *P. copri*, and only a small amount of *Prevotella stercorea* was included at the species level (Figures 1B,C). To explore the effect of dietary intervention on enterotype, we compared individual enterotype changes before and after diet intervention. After the Low-GID intervention, the ET-P proportion among 52 patients increased from 43.4 to 54.7%, and the ET-B proportion decreased from 56.6 to 45.3%. After the CD diet, the ET-B proportion decreased from 61.5 to 51.9%, whereas the ET-P proportion increased from 38.5 to 48.1% (Figure 1D). The results indicated that both diets increased the *Prevotella* enterotype.

### Secondary Outcomes: Changes in α-Diversity, β-Diversity, and Fecal Bacteria

**FIGURE 2:**
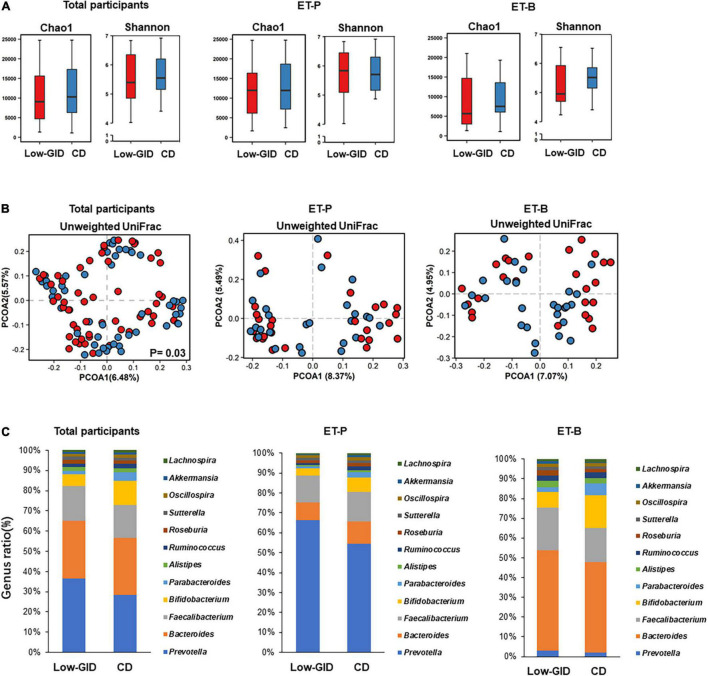
The overall difference of intestinal microbes in the *Prevotella* and *Bacteroides* intestinal clusters after the Korean traditional balanced diet (Low-GID) and the westernized Korean diet, the control diet (CD), interventions. **(A)** The α-diversity difference between Chao and Shannon indices. **(B)** PCOA based on unweighted UniFrac. **(C)** Differences in the relative abundance of intestinal microbes at the genus level. Gut microbes with a total count of less than 400 and unclassified ones were truncated. A linear mixed model for α-diversity was used for statistical analysis; a significant difference is represented by *p* < 0.05. Using AMOVA for α-diversity’s statistical analysis, there is a significant difference when *p* < 0.05.

There was no significant difference between the Chao and Shannon indices of α-diversity in the participants with Low-GID and CD in all participants (Figure 2A; *p* > 0.05). The Chao and Shannon indices that represent α-diversity did not significantly differ between the Low-GID and CD in each ET-P and ET-B cluster. The PCoA chart for β-diversity calculated from the weighted-UniFrac distance method showed significant differences in fecal bacteria between Low-GID and CD in the AMOVA in all participants (*p* < 0.05; Figure 2B). However, there was no significant difference in β-diversity between the Low-GID and CD in the ET-P and ET-B clusters (*p* > 0.05; Figure 2B).

To compare the fecal bacterial differences between the Low-GID and CD at the genus level, we intercepted the total counts of more than 400 bacterial genera. In all participants, the Low-GID exhibited a higher abundance of *Prevotella* and *Faecalibacterium* than CD, whereas CD intervention showed a higher abundance of *Bifidobacterium* and *Parabacteroides* than the Low-GID (Figure 2C). However, the differences in fecal bacteria at the genus level were not significantly different between the Low-GID and CD groups. It was due to the interaction of the participants’ enterotypes with the dietary intervention to modulate fecal bacteria differently. In the ET-P cluster, the Low-GID intervention exhibited a higher abundance of *Prevotella*, whereas the CD group had a higher relative abundance of *Bacteroides* and *Bifidobacterium* (Figure 2C). However, there were no changes in the relative abundance of *Prevotella* between Low-GID and CD groups in the ET-B cluster, whereas Low-GID increased the relative abundance of *Bacteroides* and *Faecalibacterium* but decreased that of *Bifidobacterium* (Figure 2C). Therefore, Low-GID and CD modulate the fecal bacterial community differently, whereas Low-GID increases *Bacteroides* and *Faecalibacterium* but suppresses *Bifidobacterium*.

The correlation between serum concentrations of SCFA and fecal bacteria in the genus level is shown in Supplementary Figure 1. Although serum butyric acid concentration was higher in CD, the Low-GID showed a higher positive association of fecal bacteria with serum butyric acid concentration and lower serum acetic acid concentration.

### A Novel Model for Low-Glycemic Diet and Control Diet From Biochemical and Anthropometric Parameters, Serum Metabolites, and Gut Microbiota Using Machine-Learning Classifier

**FIGURE 3:**
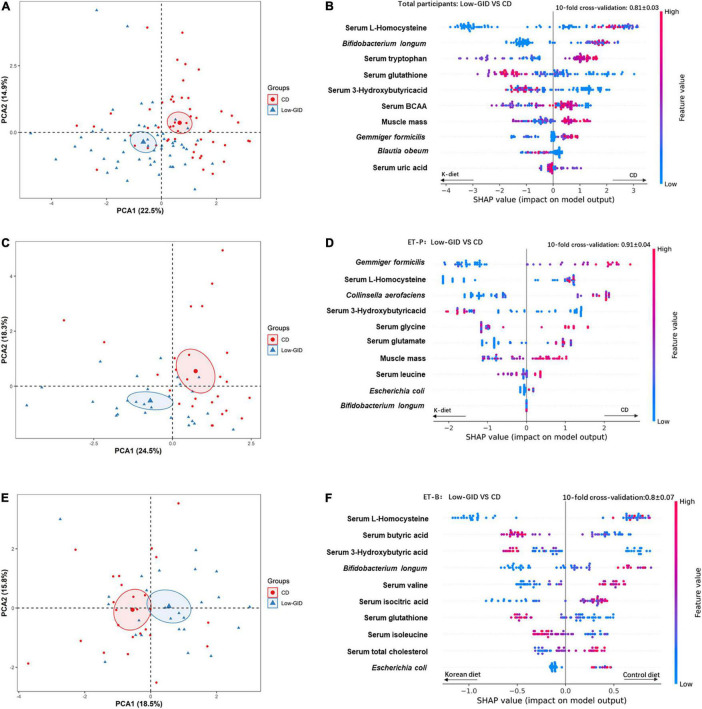
XGBoost classification model variable interpretation of Korean traditional balanced diet (Low-GID) and control diet (CD) based on the overall SHAP, *Prevotella*, and *Bacteroides* clusters. The 10-fold cross-validation is expressed in terms of mean ± SD. In the figure, the left side of the scattered dots to the 0-scale line is the Korean traditional diet classification, and the right side is the control diet classification. The height of the color represents the size of the variable. **(A)** PCA analysis of CD and Low-GID in the overall cohort. **(B)** XGBoost categorical variable interpretation, in the overall cohort. **(C)** PCA analysis of CD and Low-GID in the Prevotella enterotype cohort. **(D)** XGBoost categorical variable interpretation, in the *Prevotella* intestinal cohort. **(E)** PCA analysis of CD and Low-GID in the Bacteriodes enterotype cohort. **(F)** XGBoost categorical variable interpretation, in the *Bacteroides* intestinal cohort. BCAA, branched-chain amino acid; TG, triglyceride.

We used the relative abundance of intestinal microbes at the species level, anthropometric and biochemical parameters, and serum metabolomics as the features to train the XGBoost classification models to find a novel model to compare Low-GID and CD diet according to ET-P, ET-B, and total participants. The variables were sorted from top to bottom with the relative importance of the SHAP value to explain the Low-GID and CD effects in the novel model. After a random grid search of the features in the XGBoost algorithm, the classifiers set at max_depth = 23, n_estimators = 840, and learning_rate = 1.16. The 10-fold cross-validation accuracy was 0.81 ± 0.03, suggesting that the model had an accuracy of approximately 81% to explain the comparison of Low-GID and CD in all participants. According to the relative importance of the feature using SHAP, the model included serum L-homocysteine, *Bifidobacterium longum*, serum tryptophan, serum glutathione, serum 3-hydroxybutyric acid, serum BCAA, muscle mass, *Gemmiger formicilis*, *Blautia obeum*, and serum uric acid in total participants (Figure 3A). CD increased serum tryptophan, serum BCAA concentrations, muscle mass, and *Gemmiger formicilis* compared to Low-GID, whereas serum L-homocysteine concentrations, *Bifidobacterium longum*, and *Gemmiger formicilis* were lower than in Low-GID. However, serum glutathione and 3-hydroxybutyrate concentrations were higher in the Low-GID than the CD in all participants using the SHAP model (Figure 3A). The PCA showed the well-separation of Low-GID and CD in the SHAP model of total participants: PCA1 and PCA2 explained the variance of Low-GID and CD by 22.5 and 14.9%, respectively (Figure 3B).

The XGBoost classifier settings of the ET-P queue from the random grid search were as follows: max_depth = 84, n_estimators = 680, and learning_rate = 0.32. The 10-fold cross-validation accuracy was 0.91 ± 0.04, indicating approximately 91% accuracy of the SHAP model in the ET-P cluster. According to the relative importance of the feature using SHAP, the model included *Gemmiger formicilis*, serum L-homocysteine, *Collinsella aerofaciens*, serum 3-hydroxybutyric acid, serum glycine, serum glutamate, muscle mass, serum leucine concentrat*ions, Escherichia coli*, and *Bifidobacterium longum* in the ET-P cluster (Figure 3C). Among the ET-P cluster, CD increased *Gemmiger formicilis, Collinsella aerofaciens*, serum glycine, and muscle mass compared to the Low-GID, whereas serum L-homocysteine and serum glutamate levels decreased and serum 3-hydroxybutyric acid levels increased in the Low-GID compared to the CD using the SHAP model (Figure 3C). In the SHAP model of ET-P, Low-GID and CD showed better separation by the PCA than in the total participants: PCA1 and PCA2 explained the variance of Low-GID and CD by 24.5 and 18.3%, respectively (Figure 3D).

From the random grid search, the XGBoost classifier settings of the ET-B cluster included max_depth = 78, n_estimators = 580, and learning_rate = 0.01. The 10-fold cross-validation accuracy was 0.8 ± 0.07, suggesting that the model had approximately 80% accuracy in distinguishing the metabolic effects between the Low-GID and CD. Based on the relative importance of SHAP, the model contained serum L-homocysteine, serum butyric acid, serum 3-hydroxybutyric acid, *Bifidobacterium longum*, serum valine, serum isocitric acid, serum glutathione, serum isoleucine, serum total cholesterol concentrations, and *Escherichia coli* (Figure 3E). In the ET-B cluster, CD increased serum L-homocysteine, *Bifidobacterium longum*, serum valine, serum isocitric acid, serum total cholesterol concentrations, and *Escherichia coli* compared to the Low-GID. In contrast, serum butyric acid, serum 3-hydroxybutyric acid, serum glutathione, and serum isoleucine concentrations were higher in the Low-GID group than in the CD group (Figure 3E). In the SHAP model of ET-B, Low-GID and CD were closely clustered by the PCA: PCA1 and PCA2 explained the variance of Low-GID and CD by 18.5 and 15.8%, respectively (Figure 3F).

## Discussion

Based on community-based intestinal microbiota, extensive research has been conducted on enterotypes of different populations in different countries (26). Although most populations exhibit three enterotypes, the dominant intestinal microbial species differ due to diet and environmental reasons. *Prevotella* and *Bacteroides* are the discrete enterotypes that have been consistently identified in various studies (25–27). However, the *Ruminococcus* enterotype is not separated from the *Bacteroides* one (13). In this study, the *Ruminococcus* enterotype was not clustered as one enterotype, and all participants were divided into two discrete enterotypes, ET-B and ET-P. According to the previous studies, ET-B is related to long-term protein and animal fat intake, whereas ET-P is related to carbohydrates (11). In this study, the ET-B proportion was higher before the intervention, but the ET-P proportion increased after the intervention, which indicated that some participants changed during the intervention. The gut microbiota of adults are stabilized with age and are not easily modified (28). The participants who changed the enterotype after the intervention might have gut microbiota at the boundary of ET-P and ET-B before intervention. Previous studies have pointed out that typical long-term diet patterns do not change in adults, but only extreme dietary changes will moderate the gut microbiota (29). Therefore, short-term traditional diet interventions may not change enterotypes.

*Prevotella* is composed mainly of *Prevotella copri* (30), present in 39.1% of healthy people, especially in Asian populations (30). If present, *Prevotella copri* occupies a large part of the gut microbiota. In this study, *Prevotella copri*, at the species level of ET-P, accounted for approximately 45% of the total bacteria. In this study, the Low-GID increased *Prevotella* compared to the CD in ET-P but not ET-B, whereas the Low-GID elevated *Bacteroides* in ET-B. According to the diet, a recent study has shown that *Prevotella copri* also affects the host’s health response; the Mediterranean diet better impacts the risk of cardiometabolic diseases in people without *Prevotella.* It supports a personalized diet based on intestinal microbes (15, 31). This study also showed different impacts on fecal bacterial species and metabolism. The Low-GID reduced body weight in both ET-P and ET-B, but its decrease was related to muscle mass loss in ET-P, but not in ET-B. The Low-GID reduced serum lipid and amino acid concentrations compared to CD in both enterotypes, but the decrease was higher in ET-P than in ET-B. However, the Low-GID decreased *Bifidobacterium* compared to CD in both enterotypes. Therefore, this study showed some discrepancies in the dietary effects of ET-B and ET-P, but according to enterotypes, it is not clear which diet is better for health benefits. Therefore, further studies are needed to consider different enterotypes for personalized nutrition.

In the body composition and blood biochemical analysis, compared with CD in the two enterotypes, the Low-GID lowered BMI and maintained low serum total cholesterol and blood lipids in this study. The Low-GID and CD in the ET-P cohort showed more substantial differences in amino acids in the blood circulation than those in the ET-B group. The Low-GID group had lower serum leucine, BCAA, tryptophan, tyrosine, glycine, and L-homocysteine concentrations. According to recent studies, it has been found that there is a strong association between the chronic elevation of BCAA in serum and insulin resistance (32, 33). In a study of serum metabolites based on non-diabetic middle-aged people (34), serum tryptophan and tyrosine concentrations are found to be related to glucose intolerance and insulin resistance, and these two amino acids are also higher in patients with diabetics with decreased insulin secretion (34, 35). Serum L-homocysteine concentrations are also associated with insulin resistance and diabetes. Homocysteine interferes with the phosphorylation of insulin receptors to attenuate insulin signal transduction (36). Contrary to other serum amino acids, serum glycine concentration can reduce the accumulation of free fatty acids in the serum and is associated with a lower risk of type 2 diabetes (37). Therefore, this study suggested that the Low-GID reduced insulin resistance based on serum amino acids and their derivative concentrations compared to CD.

In addition to serum amino acids, the Low-GID group exhibited higher serum 3-hydroxybutyric acid concentrations; 3-hydroxybutyric acid is a primary ketone body produced from fatty acids, mainly under calorie restriction, a low-carbohydrate diet, and an intermittent diet. It acts as a regulatory molecule to inhibit gene expression involved in DNA methylation, oxidative stress, and inflammation (38). It contributes to increased longevity (38). The Low-GID increased serum 3-hydroxybutyrate concentrations in both enterotypes, but serum glutathione concentration increased with the Low-GID only in ET-B. This study suggests that a low-glycemic diet may have similar activity to a low-carbohydrate diet in both enterotypes. The CD group had higher serum uric acid levels than the control group. Uric acid is a product of purine metabolism in humans and other primates, and joint and soft tissue deposits can cause gout (39). The research found that serum uric acid levels are positively correlated with hyperinsulinemia and insulin resistance (40). Compared with CD, the Low-GID of the ET-B cohort had lower serum BCAA, tryptophan, and uridine levels in the serum. Uridine is associated with glucose homeostasis. Plasma uridine is a marker of insulin resistance in patients with type 2 diabetics. Animal experiments have shown that long-term or short-term uridine supplementation impairs glucose tolerance and insulin sensitivity (41). Therefore, the Low-GID could improve insulin sensitivity in both enterotypes, but its effect was more profound in ET-P than in CD.

According to the XGBoost machine learning model results, ET-P has a higher classification accuracy than ET-B. It shows the influence of the Low-GID and CD on energy and lipid metabolism in both ET-P and ET-B. In the Low-GID and CD classification, SHAP importance in all participants showed that *Blautia obeum* was positively correlated with Low-GID classification, whereas *Bifidobacterium longum* was positively linked to CD classification. *Blautia*, belonging to *Lachnospiraceae*, can reduce inflammatory and metabolic diseases, similar to probiotics (42). The classification of SHAP importance in the ET-P cohort showed that the levels of *Gemmiger formicilis*, *Collinsella aerofaciens*, and *Escherichia coli* were positively correlated with the CD classification. However, less is known about *Gemmiger formicilis*. In previous studies, *Collinsella aerofaciens* has shown a positive correlation between waist circumference and BMI (43). It corresponds to a higher BMI in the CD group. *Escherichia coli* is a widely recognized pathogenic strain (44). The SHAP importance of the ET-B cohort showed that the level of butyric acid was positively correlated with the Low-GID classification, whereas the CD group was positively correlated with serum isocitric acid concentrations. Butyric acid is an essential metabolite of intestinal microbes. Previous research has shown that butyric acid protects against obesity and obesity-related conditions and critically contributes to the diet–gut microbiota–host health axis (45). Thus, the Low-GID improved the gut microbiota community to prevent obesity and insulin resistance in ET-B. However, Low-GID decreased *Bifidobacterium*, which might be associated with milk intake.

With the advancement of nutrition and medical research, one healthy diet may not fit everyone, and it needs to be personalized according to genetics and/or gut microbiota. Each person’s response to the diet may be affected by the characteristics of the microorganisms. For instance, on the one hand, *Prevotella* is generally related to many inflammatory diseases, although it is known to be high in lean Asians who consume high amounts of vegetables and grains (30). Studies have shown that *Prevotella* is a risk factor for glucose tolerance and type 2 diabetes because it synthesizes branched-chain fatty acids (46), and it corresponds to a marked increase in type 2 diabetes in Asians. *P. copri* was also identified as the most potent driving species for the positive correlation between microbial BCAA synthesis in the gut and increased insulin resistance traits in a cohort of non-diabetic men (47). On the other hand, research supports that *P. copri* benefits glucose and insulin resistance metabolism under a diet rich in fiber (48). However, this remains controversial. In this study, Low-GID showed a metabolic benefit compared to CD, and its benefit was more evident in the ET-P in reducing many possible causes of insulin resistance and improving glucose homeostasis. For enterotypes, further large-scale clinical studies are needed to determine the effect of Low-GID on energy, glucose, and lipid metabolism.

Our study has some limitations. The experimental sample size is small for studying metabolism according to enterotypes, although the machine-learning model is especially needed for large sample sizes. By applying a machine-learning approach to large samples, we may be able to understand more precise and in-depth connections among the gut microbiota, metabolic changes, and diet. At the same time, the CD meal represents a modern western-style Korean meal, and its difference was not large enough to show the differences between diet patterns.

## Conclusion

Our results show that the Low-GID was beneficial for energy and lipid metabolism in both ET-P and ET-B clusters, but it was more pronounced in ET-P. The Low-GID improved lipid metabolism and serum metabolites related to insulin resistance in ET-P more effectively, although the Low-GID reduced muscle mass compared to CD. Low-GID can be recommended to overweight and obese women with both enterotypes, although its efficacy was more profound in ET-P. This study suggested that diet patterns could influence metabolic traits differently in obese women with different enterotypes, and the results support the possibility of a personalized diet based on enterotypes. Further studies are needed to find appropriate diets for each enterotype.

## Data Availability Statement

The datasets presented in this study can be found in online repositories. The names of the repository/repositories and accession number(s) can be found below: NCBI; PRJNA801359.

## Ethics Statement

The Institutional Review Board approved the study protocol at CHA University (1044308-201801-HR-033-04), and the study was conducted under the Helsinki Declaration. All participants provided written informed consent. The patients/participants provided their written informed consent to participate in this study.

## Author Contributions

M-SK and SP conceptualized the study. XW and SP contributed to methodology, investigation, and writing the original draft. XW, HH, and K-HL contributed to formal analysis. HY, MH, and MK contributed to validation. M-SK contributed to funding acquisition. All authors contributed to supervision, writing, reviewing, and editing the manuscript, and approved the submitted version.

## Conflict of Interest

The authors declare that the research was conducted in the absence of any commercial or financial relationships that could be construed as a potential conflict of interest.

## Publisher’s Note

All claims expressed in this article are solely those of the authors and do not necessarily represent those of their affiliated organizations, or those of the publisher, the editors and the reviewers. Any product that may be evaluated in this article, or claim that may be made by its manufacturer, is not guaranteed or endorsed by the publisher.

## Footnotes

1
https://enterotype.embl.de/enterotypes.html
