# Supplementary material for: Beneficial Effects of a Low-Glycemic Diet on Serum Metabolites and Gut Microbiota in Obese Women With Prevotella and Bacteriodes Enterotypes: A Randomized Clinical Trial
Source: Front Nutr. 2022 May 2;9:861880. doi: 10.3389/fnut.2022.861880 (PMC9111978; doi:10.3389/fnut.2022.861880)
Supplement: Supplementary file 1 [file Image_1.pdf]

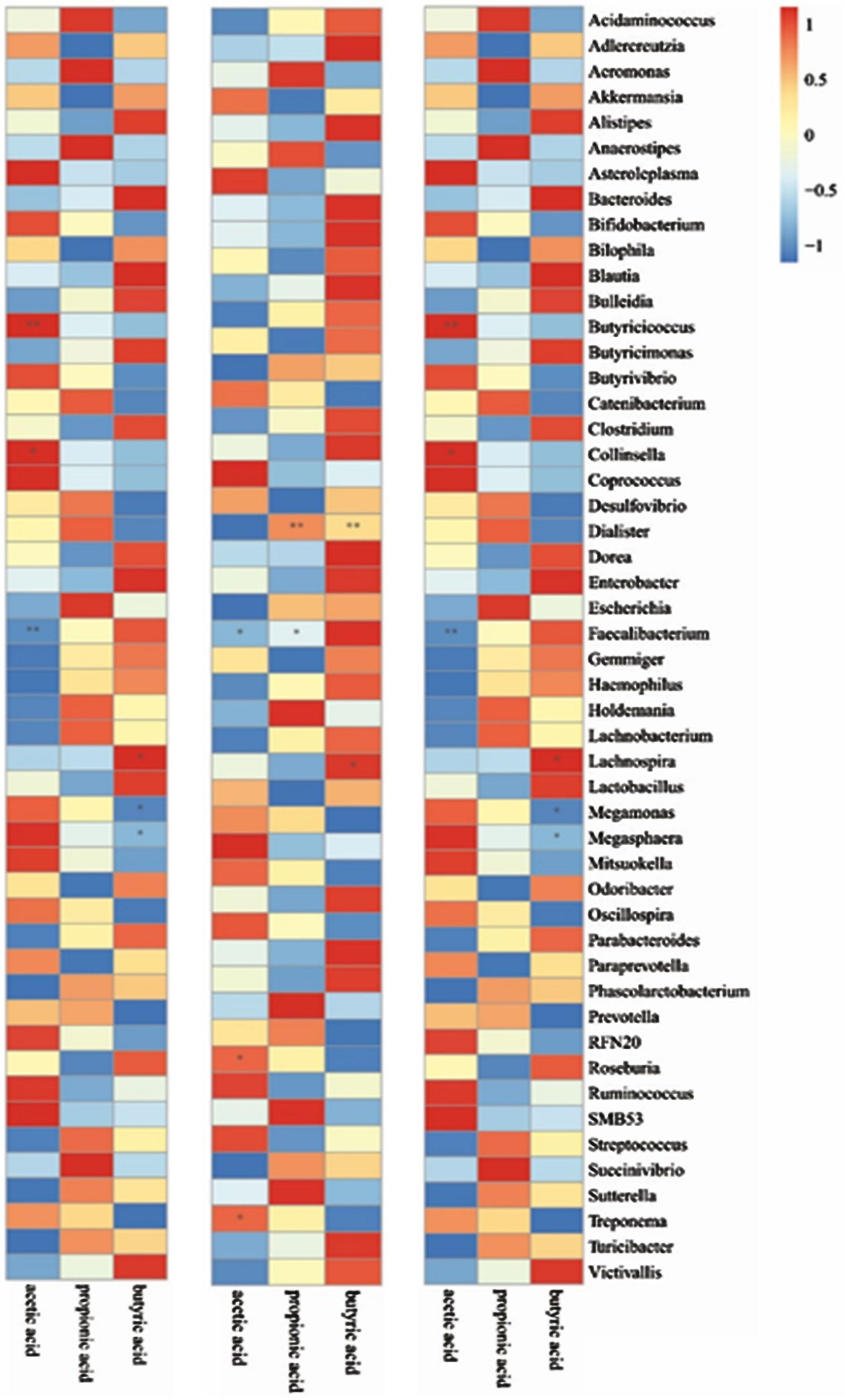

Acidaminococcus
Adlercreutzia
Aeromonas
Akkermansia
Alistipes
Anaerostipes
Asteroleplasma
Bacteroides
Bifidobacterium
Bilophila
Blautia
Bulleidia
Butyricicoccus
Butyricimonas
Butyrivibrio
Catenibacterium
Clostridium
Collinsella
Coprococcus
Desulfovibrio
Dialister
Dorea
Enterobacter
Escherichia
Faecalibacterium
Gemmiger
Haemophilus
Holdemania
Lachnobacterium
Lachnospira
Lactobacillus
Megamonas
Megasphaera
Mitsuokella
Odoribacter
Oscillospira
Parabacteroides
Paraprevotella
Phascolarctobacterium
Prevotella
RFN20
Roseburia
Ruminococcus
SMB53
Streptococcus
Succinivibrio
Sutterella
Treponema
Turicibacter
Victivallis
1
0.5
0
−0.5
−1
acetic acid
propionic acid
butyric acid
acetic acid
propionic acid
butyric acid
acetic acid
propionic acid
butyric acid

A. Total participants B. Low-GID C. CD

Supplemental Fig. 1S. Correlation between serum short-chain fatty acids (acetic acid, propionic acid, and butyric acid) concentrations and fecal bacteria at the genus level according to the diets.

* Significantly correlated with serum SCFA and fecal bacteria at $P<0.05$ ** at $P<0.01$.
